# Identification and Validation of the Pyroptosis-Related Molecular Subtypes of Lung Adenocarcinoma by Bioinformatics and Machine Learning

**DOI:** 10.3389/fcell.2021.756340

**Published:** 2021-11-04

**Authors:** Le-Ping Liu, Lu Lu, Qiang-Qiang Zhao, Qin-Jie Kou, Zhen-Zhen Jiang, Rong Gui, Yan-Wei Luo, Qin-Yu Zhao

**Affiliations:** ^1^Department of Blood Transfusion, The Third Xiangya Hospital of Central South University, Changsha, China; ^2^Department of Laboratory Medicine, The Third Xiangya Hospital of Central South University, Changsha, China; ^3^College of Engineering and Computer Science, The Australian National University, Canberra, ACT, Australia

**Keywords:** lung adenocarcinoma, pyroptosis, subtype, machine learning, prognostic

## Abstract

Lung cancer remains the leading cause of cancer death globally, with lung adenocarcinoma (LUAD) being its most prevalent subtype. Due to the heterogeneity of LUAD, patients given the same treatment regimen may have different responses and clinical outcomes. Therefore, identifying new subtypes of LUAD is important for predicting prognosis and providing personalized treatment for patients. Pyroptosis-related genes play an essential role in anticancer, but there is limited research investigating pyroptosis in LUAD. In this study, 33 pyroptosis gene expression profiles and clinical information were collected from The Cancer Genome Atlas (TCGA) and Gene Expression Omnibus (GEO) databases. By bioinformatics and machine learning analyses, we identified novel subtypes of LUAD based on 10 pyroptosis-related genes and further validated them in the GEO dataset, with machine learning models performing up to an AUC of 1 for classifying in GEO. A web-based tool was established for clinicians to use our clustering model (http://www.aimedicallab.com/tool/aiml-subphe-luad.html). LUAD patients were clustered into 3 subtypes (A, B, and C), and survival analysis showed that B had the best survival outcome and C had the worst survival outcome. The relationships between pyroptosis gene expression and clinical characteristics were further analyzed in the three molecular subtypes. Immune profiling revealed significant differences in immune cell infiltration among the three molecular subtypes. GO enrichment and KEGG pathway analyses were performed based on the differential genes of the three subtypes, indicating that differentially expressed genes (DEGs) were involved in multiple cellular and biological functions, including RNA catabolic process, mRNA catabolic process, and pathways of neurodegeneration-multiple diseases. Finally, we developed an 8-gene prognostic model that accurately predicted 1-, 3-, and 5-year overall survival. In conclusion, pyroptosis-related genes may play a critical role in LUAD, and provide new insights into the underlying mechanisms of LUAD.

## Introduction

Lung cancer is the second most prevalent cancer worldwide, and remains the leading cause of cancer death globally (Sung et al., 2021). Non-small cell lung cancer (NSCLC) accounts for 85% of lung cancers, with lung adenocarcinoma (LUAD) accounting for half of NSCLC (Herbst et al., 2008; Sivakumar et al., 2017). Despite immense progress that many therapeutic strategies have shown, the survival rate of LUAD is still low. Various blocking immune checkpoint therapies such as programmed cell death protein 1 (PD1, PDCD1) and T-lymphocyte-associated antigen 4 (CTLA4) have shown significant efficacy in the treatment of LUAD (Topalian et al., 2016). However, a proportion of LUAD patients were resistant to chemotherapy, immunotherapy, or targeted therapy, leading to cancer relapse or death (Zhang Y. et al., 2020).

The reason why these therapies failed in some patients is partly due to the heterogeneous of LUAD. Patients given the same treatment regimen may have different responses and clinical outcomes (Topalian et al., 2012). Therefore, it is significant to identify novel subtypes of LUAD, to predict prognosis and provide personalized treatment for patients.

Pyroptosis, a kind of programmed death, is morphologically characterized by cell swelling, formation of a large number of bubble-like protrusions, rupture of the plasma membrane, and release of cell contents and inflammatory factors such as IL6 and IL8 without causing mitochondrial rupture, which then activates the inflammatory response (Tan et al., 2021). Recently, it has been shown that tumor cells that die by pyroptosis produce large amounts of antigens that stimulate a systemic immune response and inhibit tumor growth (Tang et al., 2020). In addition, GSDME, a key protein for pyroptosis, can increase adaptive immunity of tumors by promoting macrophage-mediated phagocytosis (Zhang Z. et al., 2020), making it impossible for tumor cells to evade immune surveillance (Werfel and Cook, 2018; Tang et al., 2020). Immune cell infiltration is increased in tumors with high expression of GSDME, whereas GSDME-deficient tumors have reduced immune cell infiltration (Zhang Z. et al., 2020). One study knocked out pyroptosis-related genes in mice and found that these mice were prone to inflammation-associated colon cancer (Hu et al., 2010). Thus pyroptosis is an anti-cancer approach with great potential. There is only one type study related to ovarian cancer and pyroptosis (Ye et al., 2021), which demonstrated significant differences in prognosis and tumor immunity based on molecular subtypes of ovarian cancer identified by pyroptosis-related genes. No investigators have yet studied the mechanism of pyroptosis exerting anti-cancer in LUAD and its relationship with patient treatment and prognosis.

With the development of high-throughput sequencing, the understanding of tumor gene expression profiles is more comprehensive and in-depth, which will help in classifying tumors and precise treatment. Many studies have been conducted to identify subtypes, predict prognosis or predict drug resistance based on the expression of different genes and clinical features (Chen et al., 2021; Tian et al., 2021; Zhang S. et al., 2021). In this study, we did a prior study on identifying the pyroptosis-related molecular subtypes of LUAD, based on the high-throughput sequencing data. Our study explored the roles that pyroptosis play in different subtypes, and may shed light on personalized treatment for LUAD patients.

In our study, the expression profiles of LUAD patients were downloaded from the TCGA and the GEO databases, and 33 pyroptosis-related genes were obtained from a previous review. The patients were classified into three subtypes, based on the expression profile and clinical characteristics of pyroptosis-related genes in LUAD patients. 10 key pyroptosis-related genes were then selected by a machine learning algorithm, making the subtyping more convenient for clinicians to use. We explored the prognosis, the functional pathways involved in the differential genes, and the immune infiltration of each subtype. Finally, a webpage was created to facilitate clinician use.

## Materials and Methods

### Datasets

Firstly, a total of 1577 LUAD samples’ RNA sequencing (RNA-seq) data and corresponding clinical information were obtained from The Cancer Genome Atlas database (TCGA)^1^ and the Gene Expression Omnibus database (GEO)^2^, respectively (Supplementary Table 1). To ensure the reliability of survival results, we included LUAD patients from the TCGA or GEO databases with complete clinical information. As a result, 535 samples from TCGA database were served as the training cohort, and 1,042 samples from GEO database (ID: GSE31546, GSE19188, GSE30219, GSE37745, GSE50081, GSE31210, and GSE68465) were included in our study as the external validation cohort.

### Construction of Molecular Subtypes

We obtained 33 pyroptosis-related genes from prior studies (Karki and Kanneganti, 2019; Liu D. et al., 2021; Ye et al., 2021; Zhou et al., 2021), and they are presented in Supplementary Table 2. Consensus Clustering, an unsupervised clustering method, is a common cancer subtype classification method in which the clustering framework incorporates results from multiple runs of an inner-loop clustering algorithm on sub-sampled subjects (Wilkerson and Hayes, 2010; Seymour et al., 2019). The “ConsensusClusterPlus” R package was used for consensus clustering and distinguishing different molecular subtypes based on the mRNA expression data of 33 pyroptosis-related genes (Tian et al., 2021). Pairwise consensus values are calculated and stored in a consensus matrix (CM) for each k (Zheng J. et al., 2020). The empirical cumulative distribution function (CDF) plots revealed the consensus distributions for each k, and we used CDF plots and cluster-consensus plots to help choose a number of clusters and access cluster stability (Wilkerson and Hayes, 2010; Hong et al., 2021).

Next, in order to simplify the method of LUAD molecular subtype classification to improve its clinical applicability, we optimized the classification model using a machine learning approach. CatBoost is an improved implementation of gradient enhanced decision trees (GDBT) algorithm developed by Yandex. It has demonstrated excellent performance on many classification and regression tasks (Kang et al., 2021; Liu S. et al., 2021; Wang Y. et al., 2021). CatBoost was performed via the Python package to build a new optimized classification model (CatBoost model).

### Characterization of Molecular Subtypes of Lung Adenocarcinoma

Firstly, we used the entire TCGA dataset as the training set and all samples from the GEO database as the validation set. LUAD patients were classified into different molecular subtypes by using the CatBoost model based on the expression levels of key pyroptosis-related genes. Furthermore, overall survival (OS) and progression-free survival (PFS) rate were analyzed by the Kaplan–Meier method using “survival” R package, and differences between survival distributions were assessed with the log-rank test (Wei et al., 2019). In addition, t-distributed stochastic neighbor embedding (t-SNE) was employed to explore the distribution of different subtypes and estimate the classification effect using the “Rtsne” R package. Moreover, by using the “heatmap” R package, we analyzed the correlation between the expression levels of pyroptosis-related genes of different molecular subtypes and clinicopathological features, as well as the differences in the expression profiles of pyroptosis-related genes between normal and tumor tissues.

### Immune Cells Infiltration Profile and Chemotherapeutic Response Analysis

The level of immune cell infiltration in the LUAD samples was quantified by inferencing the infiltrating cells in the tumor microenvironment (TME). We applied integrated bioinformatics methods, including MCPcounter, xCell, quanTIseq, CIBERSORTx, single-sample gene set enrichment analysis (GSEA) to evaluate differences of immune status among different molecular subtypes (Finotello et al., 2019; Cai et al., 2021; Yuan et al., 2021). Furthermore, the ‘‘pRRophetic’’ R package was used to predict the chemotherapy response of each sample based on Genomics of Drug Sensitivity in Cancer (GDSC)^3^, and the correlation between molecular subtypes and immune checkpoint genes was analyzed (Geeleher et al., 2014; Kuang et al., 2021).

### Identification of Differentially Expressed N6-Methyladenosine Regulatory Genes

A total of 12 N6-methyladenosine (m6A) regulatory genes were selected (*ALKBH5, FTO, HNRNPC, METTL3, METTL14, RBM15, WTAP, YTHDC1, YTHDC2, YTHDF1, YTHDF2*, and *ZC3H13*) as classical regulators of m6A RNA methylation according to previously published studies (Feng et al., 2021; Hou et al., 2021; Wang W. et al., 2021; Zheng B. et al., 2021). We systematically compared the mRNA expression levels of these regulatory genes base on RNA-seq transcriptome data and visualized the analysis results using boxplots.

### Functional Enrichment Analysis

Firstly, a “Limma” R package was used to identify differentially expressed genes (DEGs) among different molecular subtypes in LUAD. The cutoff value was | log2FC| ≥ 1 and adjusted *P*-value < 0.01. Then based on DEGs, the biological function was analyzed by Gene ontology (GO) and Kyoto Encyclopedia of Genes and Genomes (KEGG) pathway enrichment analysis using the “clusterProfiler” R package (Pu et al., 2021). GO or KEGG pathways with adjusted *P*-value < 0.05 were considered statistically significant.

### Generation of Prognostic Signature

Next, univariate Cox proportional hazard regression analysis was utilized to assess the relationship between this DEGs and OS of LUAD patients. Only *P* < 0.05 was considered as the valuable prognostic DGEs which were sorted out to perform the Least Absolute Shrinkage and Selection Operator (LASSO) Cox regression analysis which depend on the “glmnet” R package (Kuang et al., 2021). The risk score is equal to the sum of Lasso regression coefficient of each mRNA multiplied by the normalized expression levels of each mRNA. Finally, a “rms” R package was used to construct a nomogram base on candidate predicted genes in the TCGA dataset (Zhong et al., 2021). A calibration chart is constructed to evaluate the consistency between the predicted survival probability at 1-, 3-, and 5-year by nomogram and the actual value.

## Results

### Overall Design of This Study

**FIGURE 1:**
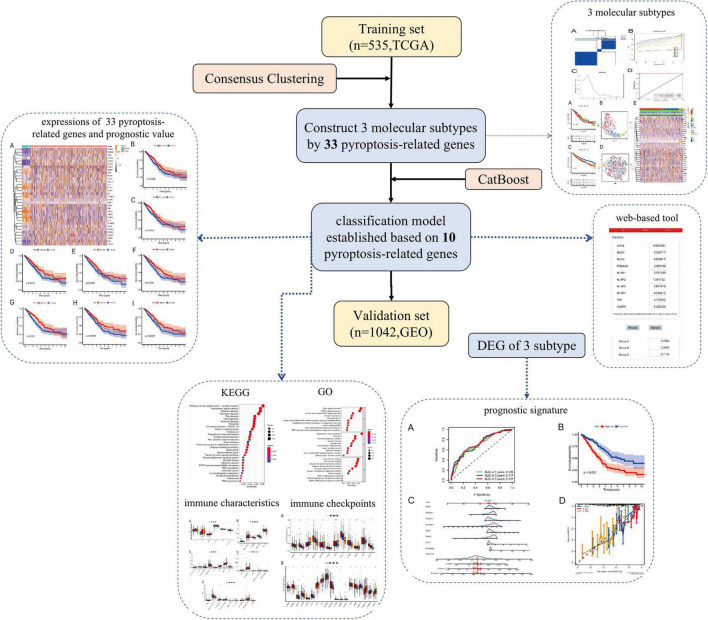
Article framework and workflow. TCGA, the cancer genome atlas database; GEO, gene expression omnibus Database; DEG, differentially expressed genes; CatBoost, categorical boosting; KEGG, kyoto encyclopedia of genes and genomes; GO, gene ontology.

Article framework and workflow have been systematically described in Figure 1. Firstly, we downloaded gene expression profiles and corresponding clinical data of LUAD patients from the TCGA database and GEO database. Samples with incomplete information were excluded. Next, LUAD patients in the TCGA dataset were divided into three different molecular subtypes (A-, B-, and C-cluster) by consensus clustering analysis based on 33 pyroptosis-related genes. Then, the machine learning method was used to simplify and optimize the judgment method of the above molecular subtypes. Based on the CatBoost algorithm, we developed a new classification model for molecular subtypes (CatBoost model). The CatBoost model included only 10 key pyroptosis-related genes with excellent classification effect. We also established a web-based tool for clinicians to use the compact model. Furthermore, we used the TCGA cohort and the GEO cohort as the training set and validation set of the model, respectively. After judging the molecular subtypes of each LUAD patient using the CatBoost model, the outcomes corresponding to the three molecular subtypes and the classification effect of the CatBoost model were explored by survival analysis and t-SNE analysis, respectively. In addition, estimation of Immune Infiltration, chemotherapeutic response prediction, expression analysis of m6A regulators and analyses of pathway enrichment were performed in different molecular subtypes of LUAD. Finally, based on molecular subtype-associated DGEs, we constructed a new prognostic signature for LUAD, and a nomogram was used to further assess the robustness and discriminatory ability of this prognostic signature.

### Identification and Verification of Molecular Subtypes of Lung Adenocarcinoma

**FIGURE 2:**
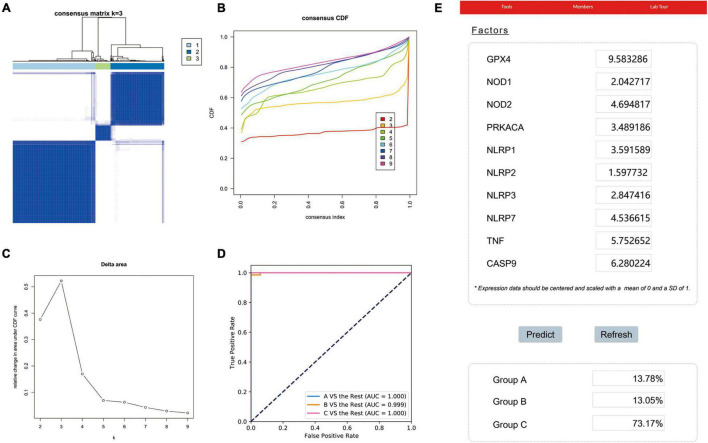
The construction of pyroptosis-related LUAD subtypes in the TCGA cohort. **(A)** Three pyroptosis-related molecular subtypes were generated via unsupervised consensus clustering. **(B)** The empirical cumulative distribution function (CDF) plots revealed the consensus distributions for each k. **(C)** The delta area score displayed the relative growth in cluster stability. A classification CatBoost model was established based on 10 key pyroptosis-related genes. **(D)** Showed receiver operating characteristic curves (ROCs) of CatBoost. **(E)** An example of the web-based tool.

To identify potential molecular subtypes of LUAD, we classified 535 LUAD patients from the TCGA cohort by using a consensus clustering analysis. In brief, the “ConsensusClusterPlus” package was applied to divide all tumor samples into k (*k* = 2–9) different subtypes according to the expression levels of 33 pyroptosis-related genes in LUAD. The results of cluster analysis showed that *K* = 3 was the optimal number of clusters, and the intragroup correlations were the highest and the intergroup correlations were low, indicating that the LUAD patients were accurately divided into three subtypes (A-, B-, and C-cluster) (Figure 2A). On the basis of the consensus scores, the CDF curve achieves the best partition efficiency when *k* = 3, and the relative change of the area under the CDF curve indicates a nearly perfect stable distribution of LUAD patients when divided into three subtypes (Figures 2B,C). In order to make the above molecular subtypes more clinically applicable, we optimized the LUAD classification prediction model using a machine learning method. Further analysis was performed by using the CatBoost algorithm to select the key predictors, including *GPX4, NLRP7, NLRP1, NLRP3, NLRP2, NOD1, NOD2, PRKACA, TNF*, and *CASP9*, which were the main factors affecting the classification of LUAD patients. Furthermore, we developed a new predictive model—the CatBoost model, based on these 10 key pyroptosis-related genes, which had excellent classification prediction ability. As shown in Figure 2D, the areas under the time-dependent ROC of molecular subtypes are 1.000, 0.999, and 1.000 for A-, B-, and C-cluster, respectively, and indicated that this CatBoost model has achieved excellent partition efficiency. In addition, a web-based tool was established for clinicians to use the compact model, and an example was showed in Figure 2E^4^.

**FIGURE 3:**
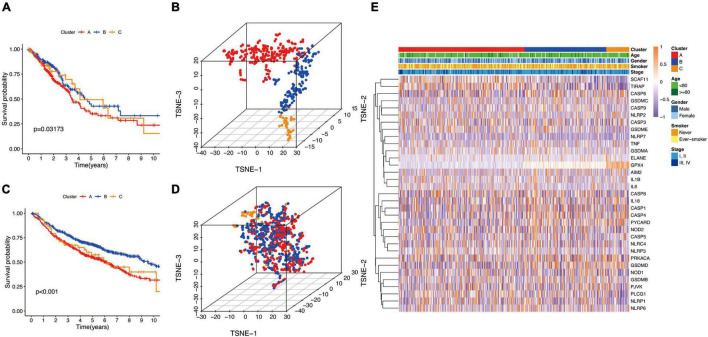
The landscape of pyroptosis-related LUAD subtypes. **(A,C)** Kaplan Meier analysis of three molecular subtypes in the TCGA and GEO cohort, respectively. **(B,D)** t-distributed stochastic neighbor embedding (t-SNE) analysis in the TCGA and GEO cohort, respectively. **(E)** Heat map of the expression profile of pyroptosis-related genes and the distribution of clinicopathological parameters in all three subtypes in TCGA cohort.

Next, we used the CatBoost model to determine which molecular subtype each of the 535 LUAD patients from the TCGA cohort belonged to. The survival analysis was performed by the Kaplan–Meier method using “survival” R package, and the results showed that significant differences in survival outcomes of different subtypes and the survival outcome corresponding to B-cluster was better, while the survival outcome of C-cluster was the worst (*P* = 0.032) (Figure 3A). The t-SNE analysis using a “Rtsne” R package showed the excellent partition efficiency when *k* = 3, indicating the robust and reliable clustering of the samples (Figure 3B). Furthermore, we used 1042 LUAD patients from the CEO database as a validation cohort to further assess the robustness and reliability of the classification model. Similarly, we first classified LUAD patients from the GEO database with the CatBoost model, and then verified the effect of classification by survival analysis and t-SNE analysis. As shown in Figure 3C, a similar result was validated in the GEO cohort, and the survival outcome of B-cluster was better (*p* < 0.001). Although, t-SNE analysis showed that A- and B-cluster were not well distinguished, C-cluster, the molecular subtype with the worst outcome, was distinguished (Figure 3D). Furthermore, the correlation between gene expression profiles and the distribution of clinicopathological parameters including age (≤80 or >80 years), gender (male or female), smoker (never or ever-smoker) and stage (I, II or III, IV) in each subtype was showed in Figure 3E. We found differences in the expression profiles of pyroptosis-related genes between different molecular subtypes. However, there was no obvious correlation between molecular subtypes and clinicopathological features of LUAD patients.

### Differential Expression and Prognostic Value of Pyroptosis-Related Genes

**FIGURE 4:**
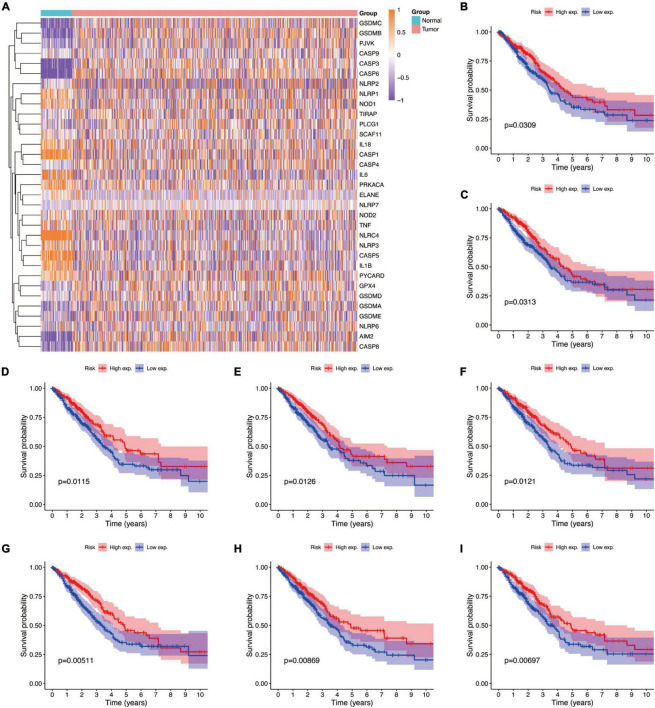
The expressions of the 33 pyroptosis-related genes and prognostic value for LUAD. **(A)** Heatmap (blue, low expression level; orange, high expression level) of the pyroptosis-related genes between the normal and the tumor tissues. **(B–I)** Kaplan–Meier analysis of *GPX4, NLRC4, NLRP1, NLRP2, NLRP3, NOD1, PLCG1*, and *PRKACA* in TCGA cohort. Adjusted *P*-value < 0.05 is considered significant (High exp., high expression level; Low exp., low expression level).

Studies have shown that the expression levels of pyroptosis-related genes affect a variety of biological processes in cancer cells and are associated with cancer development and progression. Cancers are highly heterogeneous diseases, and there are differences in the expression of certain genes. Thus, we performed differential expression analysis of pyroptosis-related genes subsequently base on mRNA expression data of LUAD in TCGA database by using a “Limma” R package, with the criterion of | log2FC| ≥ 1 and adjusted *P*-value < 0.01. The expression profiles of pyroptosis-related genes were showed in Figure 4A, and we could visually see that there were differences in the expression of pyroptosis-related genes between tumor and normal tissues. Moreover, Kaplan–Meier analysis results revealed that a higher expression of *GPX4, NLRC4, NLRP1, NLRP2, NLRP3, NOD1, PLCG1*, and *PRKACA* was associated with a better OS (*P* < 0.05) (Figures 4B–I).

### Estimation of Immune Cell Infiltration

**FIGURE 5:**
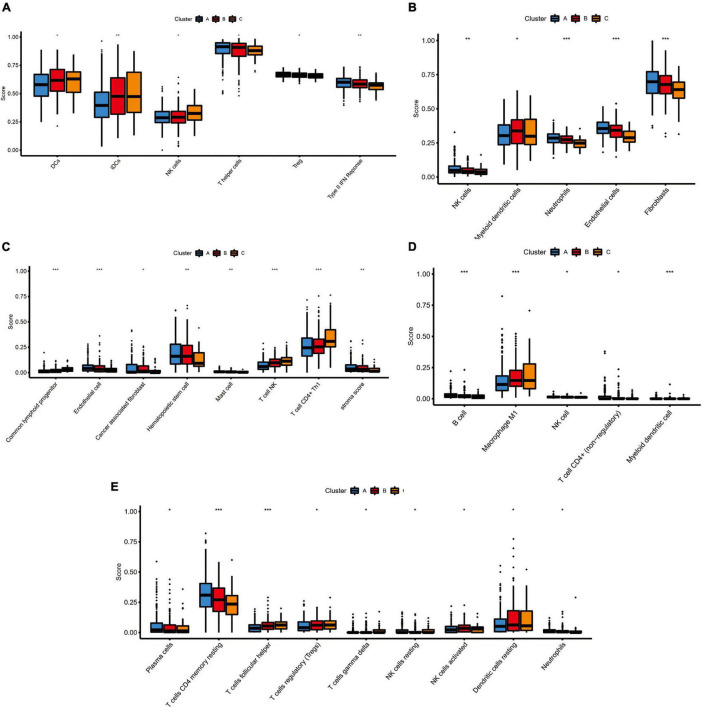
The immune landscape of three pyroptosis-related molecular subtypes. **(A)** The results of single-sample gene set enrichment analysis (ssGSEA): boxplots of ssGSEA scores among three molecular subtypes. **(B)** The results of MCPcounter analysis: boxplots comparing the abundances of immune cell subsets among three molecular subtypes. **(C)** The results of xCell analysis: boxplots comparing the abundances of immune cell subsets among three molecular subtypes. **(D)** The results of quanTIseq analysis: boxplots comparing the abundances of immune cell subsets among three molecular subtypes. **(E)** The results of CIBERSORTx analysis: boxplots comparing the abundances of immune cell subsets among three molecular subtypes. Adjusted *P*-values were showed as: ns, not significant; *P < 0.05; **P < 0.01; ***P < 0.001.

Because TIM is important for the development, progression, and treatment of cancer, we further explored the differences on immune characteristics among the three molecular subtypes. We utilized multiple immune deconvolution methods, including MCPcounter, xCell, quanTIseq, CIBERSORTx, and ssGSEA algorithms, to quantify the infiltration scores of immune cells and immune-related functions among the three molecular subtypes. As shown in Figure 5, all the analysis results revealed that there were significant differences in infiltration degree of immune cell. The ssGSEA analysis showed significant differences of infiltration degree in DCs (*P* < 0.05), iDCs (*P* < 0.01), NK cells (*P* < 0.05), T helper cells (*P* < 0.05), and Treg (*P* < 0.05). In addition, an immune function score of Type II IFN Response showed a significant difference among the three molecular subtypes (*P* < 0.01) (Figure 5A). For the MCPcounter analysis, we found differences in the infiltration of NK cells (*P* < 0.01), Myeloid dendritic cells (*P* < 0.05), Neutrophils (*P* < 0.001) and endothelial cells (*P* < 0.001), fibroblasts (*P* < 0.001) among different molecular subtypes, and the correlations between MCPcounter scores and different molecular subtypes groups showed that C-cluster had the lowest level of immune cell infiltration, suggesting a low immune response to C-cluster, consistent with its poor outcome (Figure 5B). For xCell method, the infiltration scores of common lymphoid progenitor (*P* < 0.001), endothelial cell (*P* < 0.001), cancer associated fibroblast (*P* < 0.05), Hematopoietic stem cell (*P* < 0.01), mast cell (*P* < 0.01), T cell NK (*P* < 0.001), T cell CD4 + Th1 (*P* < 0.001), and stroma (*P* < 0.01) were different among three molecular subtypes (Figure 5C). In addition, the infiltrating immune cells were also estimated by quanTIseq method, and we obtained 5 major immune cell subsets with different infiltration degree, including B cell (*P* < 0.001), Macrophage M1 (*P* < 0.001), NK cell (*P* < 0.05), T cell CD4+ (*P* < 0.05) and Myeloid dendritic cell (*P* < 0.001) (Figure 5D). The 22 types of infiltrating immune cells inferred by CIBERSORTx include B cells, T cells, natural killer cells, macrophages, dendritic cells, eosinophils, and neutrophils, and 9 of these types had significantly different degrees of infiltration including Plasma cells (*P* < 0.05), T cells CD4 memory resting (*P* < 0.001), T cells follicular helper (*P* < 0.001), Treg (*P* < 0.05), T cells gamma delta (*P* < 0.05), NK cells resting (*P* < 0.05), NK cells activated (*P* < 0.05), dendritic cells resting (*P* < 0.05), neutrophil (*P* < 0.05) (Figure 5E).

### Expression of N6-Methyladenosine Regulators and Prediction of Therapeutic Response to Immune Checkpoint Inhibitors

**FIGURE 6:**
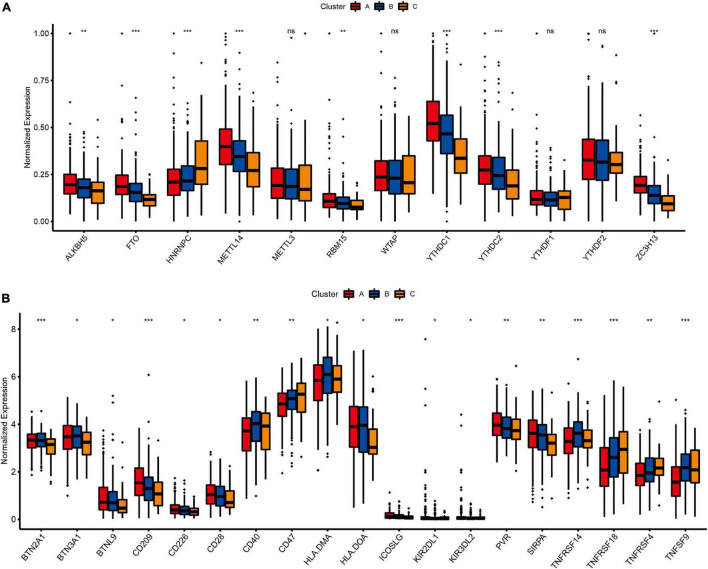
The relationship between LUAD subtypes and m^6^A regulators and immune checkpoints. **(A)** Differences in expression of m^6^A regulators among three pyroptosis-related molecular subtypes. **(B)** Differences in expression of immune checkpoint blockade genes among three pyroptosis-related molecular subtypes. Adjusted *P*-values were showed as: ns, not significant; *P < 0.05; **P < 0.01; ***P < 0.001.

As one of the most important RNA modifications, m6A is involved in regulating gene expression and physiological and pathological processes, such as tumor development and progression (Dong et al., 2021; Garbo et al., 2021; Han et al., 2021). Furthermore, m6A regulators play an important role in lung cancer progression, for example FTOH and ALKBH5 have been shown to promote the proliferation of lung cancer cells, and YTHDF2 can regulate tumor metabolism (Khan and Malla, 2021; Xu F. et al., 2021; Xu R. et al., 2021). Therefore, we further analyzed the expression levels of m6A regulatory genes in different molecular subtypes (Figure 6A). The results showed that the expression profiles of eight m6A regulators (*ALKBH5, FTO, HNRNPC, METTL14, RBM15, YTHDC1, YTHDC2*, and *ZC3H13*) were significantly different among the three different lung cancer subtypes (*P* < 0.01). Besides, immune checkpoint inhibitors have been shown to have durable efficacy in some patients with non-small cell lung cancer (NSCLC), and we investigate the relationship between subtypes and expression levels of immune checkpoint genes. As shown in Figure 6B, the analysis results showed that there were significant differences in the expression profiles of immune checkpoints among the three different molecular subtypes, Including *BTN2A1* (*P* < 0.001), *BTN3A1* (*P* < 0.05), *BTNL9* (*P* < 0.05), *CD209* (*P* < 0.001), *CD226* (*P* < 0.05), *CD28* (*P* < 0.05), *CD40* (*P* < 0.01), *CD47* (*P* < 0.01), *HLA.DMA* (*P* < 0.05), *HLA.DOA* (*P* < 0.05), *ICOSLG* (*P* < 0.001), *KIR2DL1* (*P* < 0.05), *KIR3DL2* (*P* < 0.05), *PVR* (*P* < 0.01), *SIRPA* (*P* < 0.01), *TNFRSF14* (*P* < 0.001), *TNFRSF18* (*P* < 0.001), *TNFRSF4* (*P* < 0.01) and *TNFRSF9* (*P* < 0.001), which indicated that these three molecular subtypes may respond differently to immune checkpoint inhibitors.

### Functional Enrichment Analysis and Identification of a New Prognostic Signature Base on Molecular Subtypes of Lung Adenocarcinoma

**FIGURE 7:**
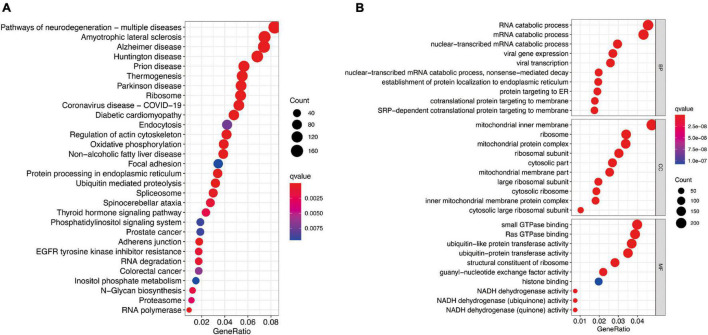
Functional analysis based on the molecular subtype-related DEGs in TCGA cohort. **(A)** Kyoto Encyclopedia of Genes and Genomes pathways. **(B)** The significant categories as determined by Gene ontology analysis. The figure represents biological process (BP), cellular component (CC), and molecular function (MF) genes from top to bottom. The bigger bubble means the more genes enriched, and the increasing depth of red means the differences were more obvious. qvalue, the adjusted *P*-value.

To further investigate potential gene functions and signaling pathways among different molecular subtypes, we extracted 5,233 DEGs in TCGA cohort (*P* < 0.01 and | log2FC| ≥ 1). Then, GO enrichment analysis and KEGG pathway analysis were performed based on these DEGs and biological processes with significant enrichment were presented in Figure 7. The analysis results showed that DEGs are involved in a variety of cellular and biological functions, including RNA catabolic process, mitochondrial inner membrane, small GTPase binding, Ras GTPase binding, ribosome, oxidative phosphorylation, protein processing in endoplasmic reticulum, and so on.

**FIGURE 8:**
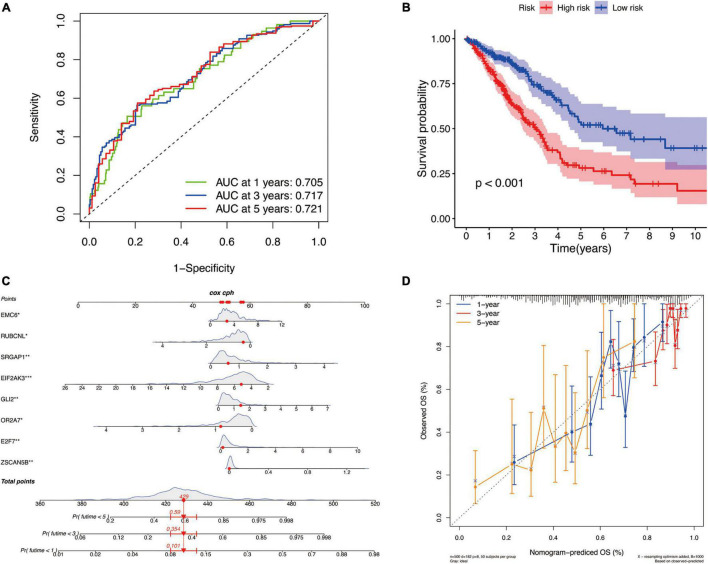
The identification of a new prognostic signature base on the molecular subtype-related DEGs for LUAD in TCGA cohort. **(A)** Areas under curves (AUCs) of the risk scores for predicting 1-, 2-, and 3-year overall survival time. **(B)** Kaplan–Meier analysis presenting difference of overall survival between the high-risk and low-risk groups. **(C)** Nomogram for predicting the probability of 1-, 3-, and 5-year overall survival time for LUAD. **(D)** Calibration plot of the nomogram for predicting the probability of overall survival at 1-, 3-, and 5-year.

There are differences in the gene expression profiles of different cancer molecular subtypes, in addition to different characteristics in tumor progression, treatment and prognosis. Therefore, based on the DEGs among these three molecular subtypes, we selected eight key DEGs (*ZSCAN5B, E2F7, OR2A7, GLI2, EIF2AK3, SRGAP1, RUBCNL*, and *EMC6*) associated with LUAD prognosis and constructed a new prognostic signature for LUAD by univariate cox regression analysis and LASSO regression algorithm. According to this prognostic signature, we used a special formula (Supplementary Calculation 3) to calculate the risk score of each sample. LUAD patients were divided into low-risk and high-risk groups based on the median of risk score. The area under the ROC curve (AUCs) were 0.705, 0.717, and 0.721 for 2-, 3-, and 5-year survival times, respectively, which demonstrated that this gene signature has a good performance in prediction for the survival of LUAD (Figure 8A). The Kaplan–Meier analysis presented difference of OS between the high-risk and low-risk groups (Figure 8B). Subsequently, to quantitatively predict the survival probability of each LUAD patient, a nomogram for 1-, 3-, and 5-year survival rates was plotted based on the gene signature risk model in TCGA database (Figure 8C). Moreover, the calibration curve result showed a high consistency in the probability of 1-, 3-, and 5-year overall survival between the nomogram prediction and the actual observation (Figure 8D).

## Discussion

In this study, 33 pyroptosis gene expression profiles and clinical information were collected from the TCGA and GEO datasets. Through a series of bioinformatics and machine learning analyses, we identified novel subtypes of LUAD based on 10 pyroptosis-related genes and further validated them in the GEO dataset, with machine learning models performing up to an AUC of 1 for classifying in GEO. We classified LUAD patients into three subtypes (A-, B-, and C-cluster), and survival analysis showed that B- had the best survival outcome and C- had the worst survival outcome. We then further analyzed the relationship between pyroptosis gene expression and clinical characteristics in the three molecular subtypes. Immune profiling revealed significant differences in immune cell infiltration among the three molecular subtypes. We further performed GO enrichment analysis and KEGG pathway analysis based on the differential genes of the three subtypes, and the results showed that DEG is involved in multiple cellular and biological functions, including RNA catabolic process, mRNA catabolic process, pathways of neurodegeneration-multiple diseases, and other functions and pathways. Finally, we developed an 8-gene prognostic model that accurately predicted overall survival at 1, 3, and 5 years in high- and low-risk groups. In conclusion, these results provide new insights into the underlying mechanisms of LUAD.

LUAD is clinically challenging due to the difficulty of early diagnosis, the ease of tumor recurrence, and poor prognosis. Studies have shown that pyroptosis is a potential therapeutic target for a variety of diseases, including infectious diseases and cancer (Fang et al., 2020). Although many prognostic-based signature bioinformatics studies of LUAD have made significant progress, the existing molecular subtype-specific identification methods are still limited. The subtyping of LUAD based on pyroptosis can provide a more comprehensive understanding of LUAD and can fill the gap of LUAD bioinformatics studies. In addition, pyroptosis affects the tumor microenvironment and tumor immunotherapy. Studying the role of pyroptosis in LUAD will help to identify potential therapeutic targets for different subtypes. In LUAD, a novel pyroptosis-related gene signature has been identified to predict prognosis (Lin et al., 2021). However, the role of a pyroptosis-related gene signature in LUAD has not been elucidated, and our study aims to elucidate this role. Our analysis of LUAD patients provides an alternative line of research and complements current bioinformatics research insights into LUAD.

In this study, further analysis using the CatBoost algorithm in machine learning identified *GPX4, NLRP7, NLRP1, NLRP3, NLRP2, NOD1, NOD2, PRKACA, TNF*, and *CASP9* as key genes affecting the classifying of LUAD. It has been found that GPX4 is closely related to tumor size and classifying in LUAD, and the higher the malignancy the lower the degree of lipid peroxidation of the tumor is. And CREB can bind to the promoter region of GPX4 to inhibit lipid peroxidation, so GPX4 may be a new target for the treatment of LUAD (Wang Z. et al., 2021). NLR family is an inflammation-related family, and they contain a caspase recruitment domain (CARD). NLR plays a key role in apoptosis, cell autophagy, inflammation, and cancer, but its relationship with pyroptosis needs to be further investigated. NLR family contains 6 of 10 key pyroptosis genes *(NLRP7, NLRP1, NLRP3, NLRP2, NOD1*, and *NOD2*), where one study found that LUAD with low NLRP1 expression had low immune cell infiltration and a poorer prognosis (Shen et al., 2021), and in another study, it was also found that LUAD patients with low expression of NLRP7, NLRP1, NLRP2, NOD1 had a poorer prognosis. It was also found that polymorphisms of NOD1/CARD4 may affect the diagnosis and treatment of lung cancer (Ozbayer et al., 2015), while polymorphisms of NOD2 were also associated with an increased risk of lung cancer (Liu J. et al., 2014). Wang Y. et al. (2016) found that activation of NLRP3 inflammasomes by LPS + ATP enhanced the proliferation and migration of A549 cells and that NLRP3 inflammasomes play a crucial role in regulating the proliferation and migration of A549 cells, and it may be a potential target for the treatment of lung cancer. The aberrant fusion of PRKACA with DNAJB1 produces a protein called PKA-CDNAJB1, a protein that has been suggested to be a major driver of fibrous lamellar hepatocellular carcinoma and is associated with other tumors whose molecular mechanisms are unclear (Olivieri et al., 2021). TNF is the promoter of pyroptosis, and it has been demonstrated that the TNF-α/HMGB1 inflammatory signaling pathway plays an important role in pyroptosis during acute liver failure and acute kidney injury (Nagashima et al., 2020). Previous studies showed that Caspase 6 plays an important role in apoptosis, and it was demonstrated that Caspase 6 can mediate innate immunity and activation of the inflammatory vesicle, and also promote activation of programmed cell death pathways, including activation of pyroptosis. In addition, Caspase 6 also plays an important role in infectious diseases and cancer (Zheng M. et al., 2020).

The regulation of inflammatory responses caused by pyroptosis is crucial in the tumor microenvironment. The present study showed significant differences in the degree of immune cell infiltration in the three subtypes, particularly NK cells, CD4 + Th1, macrophage M1, B cells, myeloid dendritic cells, and common lymphoid progenitor cells. In addition, immune function scores for type II interferon response differed significantly among the three molecular subtypes. The correlation between the MCP counter scores and the different molecular subtype groups indicated that the C-cluster had the lowest level of immune cell infiltration, indicating a low immune response to the C-cluster, consistent with its poor outcome. This indicates that the immune response differs among subtypes and reflects that our subtypes can be used as a prognostic indicator of the response to immunotherapy. Also, the analysis of the expression levels of m6A regulatory genes and immune checkpoint genes in different molecular subtypes showed different clinical responsiveness and heterogeneity of LUAD patients in different clusters. We further explored the relevant signaling pathways involved in each subtype of DEGs, and the results of KEGG and GO analysis indicated that DEGs are involved in a variety of cellular and biological functions, including RNA catabolic processes, mitochondrial endosomes, small GTPase binding, Ras GTPase binding, ribosomes, oxidative phosphorylation, and protein processing in the endoplasmic reticulum. In addition to the analysis of each of these subtypes, we have also established a nomogram for predicting prognosis at 1, 3, and 5 years by differential genes, which helps clinicians to further understand the prognosis of patients.

In this study, LUAD patients were clustered based on pyroptosis genes, and 10 key genes could be used as markers for subtyping. However, the present study also has some limitations. Initially, we only used the datasets from the online databases TCGA and GEO for the analysis, and more patient data from different regions are needed for validation. Moreover, because the dataset was derived from databases and lacked data on response to treatment, further analysis of the response to treatment of the three different subtypes is needed to help guide clinical treatment. Ultimately, the functions and pathways involved in the pyroptosis we identified, and how they regulate immune cell infiltration, need further molecular biology experiments to validate.

In summary, this study clustered LUAD based on 33 pyroptosis genes with three different subtypes (A-, B-, and C-cluster). The model was then simplified to 10 pyroptosis genes based on a machine learning approach. And a web tool was created to help clinicians use it. Survival analysis and immune characterization revealed that C-cluster had the worst survival outcome and the least immune cell infiltration. This study contributes to the understanding of the underlying molecular mechanisms of LUAD and provides some suggestions for the individualized treatment of tumor patients. The subtypes of LUAD based on pyroptosis genes may help to guide clinical treatment, assess prognosis, and predict the efficacy of immunotherapy.

## Data Availability Statement

Publicly available datasets were analyzed in this study. This data can be found here: https://portal.gdc.cancer.gov/ and https://www.ncbi.nlm.nih.gov/geo/.

## Author Contributions

L-PL, LL, and QY-Z performed the study concept and design. RG and YW-L revised the manuscript and make final approval of the version. LP-L and LL analyzed the data and wrote the manuscript. QY-Z and QJ-K helped to write the manuscript. All authors contributed to the article and approved the submitted version.

## Conflict of Interest

The authors declare that the research was conducted in the absence of any commercial or financial relationships that could be construed as a potential conflict of interest.

## Publisher’s Note

All claims expressed in this article are solely those of the authors and do not necessarily represent those of their affiliated organizations, or those of the publisher, the editors and the reviewers. Any product that may be evaluated in this article, or claim that may be made by its manufacturer, is not guaranteed or endorsed by the publisher.

## Footnotes

1
https://portal.gdc.cancer.gov/

2
https://www.ncbi.nlm.nih.gov/geo/

3
https://www.cancerrxgene.org/

4
http://www.aimedicallab.com/tool/aiml-subphe-luad.html
