# Supplementary material for: Identification and Validation of the Pyroptosis-Related Molecular Subtypes of Lung Adenocarcinoma by Bioinformatics and Machine Learning
Source: Front Cell Dev Biol. 2021 Nov 4;9:756340. doi: 10.3389/fcell.2021.756340 (PMC8599430; doi:10.3389/fcell.2021.756340)
Supplement: Supplementary file 1 [file Table_1.DOCX]

Table S1

Clinical characteristics of the three cohorts included in this study

|  |  | **The Discovery Set** | **The Validation Set** | | | | | | |
| --- | --- | --- | --- | --- | --- | --- | --- | --- | --- |
|  |  | **TCGA** | **GSE19188** | **GSE31210** | **GSE30219** | **GSE31546** | **GSE37745** | **GSE50081** | **GSE68465** |
| **LUAD Samples** |  | 535 | 40 | 226 | 85 | 16 | 106 | 127 | 442 |
| **Age, mean (SD)** |  | 65.56 (10.09) | - | 59.58 (7.40) | 61.49 (9.28) | - | 62.94 (9.22) | 68.73 (9.71) | 64.39 (10.09) |
| **Gender, n (%)** | **Male** | 249 (46.54) | 25 (62.50) | 105 (46.46) | 66 (77.65) | 3 (18.75) | 46 (43.40) | 65 (51.18) | 223 (50.45) |
| **Ever-smoker, n (%)** |  | - | - | 111 (49.12) | - | 12 (75.00) | - | 92 (72.44) | 300 (67.87) |
| **Stage, n (%)** | **I** | 294 (55.79) | - | 168 (74.34) | 71 (83.53) | - | 70 (66.04) | 92 (72.44) | - |
|  | **II** | 123 (23.34) | - | 58 (25.66) | 13 (15.29) | - | 19 (17.92) | 35 (27.56) | - |
|  | **III** | 84 (15.94) | - | 0 (0.00) | 1 (1.18) | - | 13 (12.26) | 0 (0.00) | - |
|  | **IV** | 26 (4.93) | - | 0 (0.00) | 0 (0.00) | - | 4 (3.77) | 0 (0.00) | - |
| **T, n (%)** | **1** | 175 (32.71) | - | - | 71 (83.53) | 11 (68.75) | - | 43 (33.86) | 150 (33.94) |
|  | **2** | 289 (54.02) | - | - | 12 (14.12) | 5 (31.25) | - | 82 (64.57) | 251 (56.79) |
|  | **3** | 49 (9.16) | - | - | 2 (2.35) | 0 (0.00) | - | 2 (1.57) | 28 (6.33) |
|  | **4** | 19 (3.55) | - | - | 0 (0.00) | 0 (0.00) | - | 0 (0.00) | 11 (2.49) |
|  | **X** | 3 (0.56) | - | - | 0 (0.00) | 0 (0.00) | - | 0 (0.00) | 0 (0.00) |
| **N, n (%)** | **0** | 348 (65.17) | - | - | 82 (96.47) | 15 (93.75) | - | 94 (74.02) | 299 (67.65) |
|  | **1** | 95 (17.79) | - | - | 3 (3.53) | 1 (6.25) | - | 33 (25.98) | 87 (19.68) |
|  | **2** | 74 (13.86) | - | - | 0 (0.00) | 0 (0.00) | - | 0 (0.00) | 53 (11.99) |
|  | **3** | 2 (0.37) | - | - | 0 (0.00) | 0 (0.00) | - | 0 (0.00) | 0 (0.00) |
|  | **X** | 15 (2.81) | - | - | 0 (0.00) | 0 (0.00) | - | 0 (0.00) | 1 (0.23) |
| **M, n (%)** | **0** | 361 (68.24) | - | - | 85 (100.00) | - | - | 127 (100.00) | - |
|  | **1** | 25 (4.73) | - | - | 0 (0.00) | - | - | 0 (0.00) | - |
|  | **X** | 143 (27.03) | - | - | 0 (0.00) | - | - | 0 (0.00) | - |
