# Supplementary material for: Identification and Validation of the Pyroptosis-Related Molecular Subtypes of Lung Adenocarcinoma by Bioinformatics and Machine Learning
Source: Front Cell Dev Biol. 2021 Nov 4;9:756340. doi: 10.3389/fcell.2021.756340 (PMC8599430; doi:10.3389/fcell.2021.756340)
Supplement: Supplementary file 3 [file Table_3.DOCX]

**Calculation S3**| Calculations for # risk score：

The risk score was calculated for each sample using the following formula:

Risk Score= 1.35299×ZSCAN5B + 0.18929×E2F7 + -0.50357×OR2A7 +

0.21535×GLI2 + -0.11685×EIF2AK3 + 0.39199×SRGAP1 +

-0.30320×RUBCNL + 0.08238×EMC6
